# Multi-Response Optimization of High-Performance Low-pH Grouting Materials by Using Taguchi-Based Grey Relational Analysis

**DOI:** 10.3390/ma16103891

**Published:** 2023-05-22

**Authors:** Zengzeng Ren, Weiquan Zhao, Ju Wang, Jinjie Zhang, Liang Chen, Yonghui Li

**Affiliations:** 1 State Key Laboratory of Simulation and Regulation of Water Cycle in River Basin, China Institute of Water Resource and Hydropower Research, Beijing 100038, China; rzziwhr@163.com (Z.R.); zhangjj@iwhr.com (J.Z.); leeli2011@163.com (Y.L.); 2 Beijing Research Institute of Uranium Geology, Beijing 100029, China; wangju9818@163.com (J.W.); chenliang@briug.cn (L.C.); 3 CAEA Innovation Center for Geological Disposal of High-Level Radioactive Waste, Beijing 100029, China

**Keywords:** low-pH grouting materials, microfine cement, silica fume, aluminum sulfate, slurry performance, orthogonal test, Taguchi–Grey relational analysis

## Abstract

The most accepted approach to sealing in high-level radioactive waste repositories (HLRWs) is to develop a low-pH grouting material with a pH of the pore solution of less than 11. Currently, the most widely used binary low-pH grouting material is MCSF64, which comprises 60% microfine cement (MC) and 40% silica fume (SF). In this study, a high-performance MCSF64-based grouting material was developed by incorporating naphthalene superplasticizer (NSP), aluminum sulfate (AS), and united expansion agent (UEA) to enhance the slurry’s shear strength, compressive strength, and hydration process. Orthogonal experiments were conducted to measure the flow time, yield stress, plastic viscosity, initial setting time, shear strength, and compressive strength of the MCSF64-based slurry, and the optimal mix proportion was determined using the Taguchi–Grey relational analysis method. The pH variation of the pore solution, shrinkage/expansion, and hydration products of the optimal hardened slurry were evaluated using simplified ex-situ leaching (S-ESL), a length comparometer, and scanning electron microscopy (SEM), respectively. The results demonstrate that the Bingham model effectively predicted the rheological properties of the MCSF64-based slurry. The optimum ratio for the MCSF64-based slurry was water/binder (W/B) ratio of 1.4, and the contents of NSP, AS and UEA by mass of binder were 1.9%, 3.6% and 4.8%, respectively. The optimal mix exhibited a pH value below 11 after curing for 120 days. The addition of AS and UEA facilitated hydration, shortened the initial setting time, improved early shear strength, and enhanced the expansion ability of the optimal mix under water curing conditions.

## 1. Introduction

Geological disposal has emerged as a leading strategy for long-term nuclear waste disposal in numerous countries, including Europe, the United States, China, and Japan [1]. In constructing a high-level radioactive repository (HLRW), ordinary Portland cement (OPC)-based materials are commonly utilized for various purposes, such as fissure sealing, rock bolting, tunnel and drift lining, and sealing plugs, in addition to the construction of auxiliary structures required for the repository’s operation [2,3]. In engineered barrier systems (EBSs), bentonite, a type of clay consisting mainly of montmorillonite, plays a crucial role owing to its exceptional swelling and adsorbing abilities, which effectively restrict nuclide migration. However, upon closure, the repository becomes saturated with groundwater. When OPC-based materials contact with groundwater, they generate a pore solution with a pH range of 12.5–13.8 (pH > 13 for fresh OPC-based materials, pH of 12.5 for evolved ones in equilibrium with portlandite). Such a highly alkaline plume can potentially impair bentonite’s buffering and sealing performance, thereby adversely affecting the long-term stability of the HLRW [4]. Currently, the predominant method used to preserve the stability of bentonite, which is heavily influenced by the pH of the materials it interacts with, is to produce cementitious materials that generate a pore solution with a pH of ≤11, as degradation of the bentonite buffer and host rock is restricted at or below this threshold [5,6,7].

Substituting cement with mineral admixtures such as fly ash (FA), blast furnace slag (BFS), metakaolin (MK), and silica fume (SF) at binary, ternary or even quaternary levels has emerged as an effective approach to reduce the pH of the pore solution [8]. Of these mineral admixtures, SF is recognized as the most promising agent for pH reduction in hardened cement pastes or slurry owing to its high pozzolanic activity [9]. Adding SF to cement has a dual effect, serving as both a chemically inert filler and a pozzolan that reacts with Ca(OH)_2_, and the blending of OPC with the high replacement of SF resulted after longer hydration times in the entire consumption of Ca(OH)_2_ with ettringite, and calcium silicate hydrate (C-S-H) gel with a reduced Ca/Si ratio as the only hydrate phase observed [10]. Several investigations have been conducted to identify the optimal content of SF in low-pH cementitious materials. Kim et al. [11] found that the required pH (pH ≤ 11) is obtained after curing for 130 days when the grouting material contains 40% SF. Coumes et al. [12] stated that blends containing at least 40% SF could achieve pH values below 11. Calvo et al. [13] indicated that at least 55% of SiO_2_ in the binder was needed if OPC was to be used as the basic cement of the low-pH blend, or pozzolan blended additions above 40% had to be employed. Lothenbach and Rentsch [14] studied the low-alkali cement “ESDRED” consisting of 60% CEMI and 40% SF, and found that a pH value of 11 would be reached even if the SF had reacted, as C-S-H phases would likely be stable, while ettringite would most likely convert to thaumasite in the long run. Savage and Benbow [15] suggested that SF should comprise at least 40 wt. % of dry materials to meet the pH requirement, and the Ca/Si molar ratio of C-S-H gel should be under 0.8. Based on these investigations, a 40% SF content appears to be the critical content of binary low-pH cementitious materials for achieving a target pore solution pH of ≤11.

Grouting technology has been around for hundreds of years, used to enhance engineering quality in different field applications. In terms of grouting technology, the efficacy of the grouting is highly dependent on the selection of an appropriate grouting material. Conventional grouting materials mainly include cement-based slurries, chemical slurries and organic–inorganic composite slurries. In recent years, some friendly novel composite slurries, such as geopolymer grouts and controlled low-strength materials (CLSMs), have been widely considered [16,17]. However, the slurries mentioned above are unsuitable for use due to the necessity of preventing water seepage and minimizing the damage caused by highly alkaline pore water to bentonite. For the construction of HLRW, the grouting material must possess low pH, low hydraulic conductivity, good penetrability of micro-fractures, and physical and chemical compatibility with the host environment to ensure long-term operational safety. As a result, a low-pH microfine cement (MC) material has been rapidly developed and utilized for fractures with an aperture of more than 100 μm [18,19]. Several projects have been conducted since 2002 to investigate low-pH grout, including those carried out by the Swedish Nuclear Fuel and Waste Management Company (SKB), the Nuclear Waste Management Expert of Finland (POSIVA), and the Nuclear Waste Management Organization of Japan (NUMO). Based on these studies, the MCSF64 (60% MC + 40% SF) with high W/B ratios (1.2~3.0) slurry was selected as a low-pH injection grout mix, and tested both in the laboratory and in field settings [20]. In addition, the Posiva research report emphasizes that naphthalene superplasticizers (NSPs) have a lower likelihood of complexing with nuclide ions compared to other types of water-reducing agents, hence it is recommended to use NSPs in the MCSF64 slurry for better fluidity [21]. Indeed, Holt et al. [22] investigated the properties between MCSF64 low-pH grout and plain MC grout at equivalent W/B ratios. The test results show that the former grout achieved superior performance in stability, homogeneity, and penetration ability. 

In summary, the above works have significantly contributed to improving and implementing MCSF64 low-pH grouts in HLRW. However, certain issues, including prolonged setting time, poor early shear strength, and dimensional instability, have been identified with this specific grout mix, resulting in reduced effectiveness and workability during practical grouting [11,22,23]. Therefore, the objective of this paper is to develop a slurry for microfracture grouting based on MCSF64 by incorporating NSP, aluminum sulfate (AS), and united expansion agent (UEA) to enhance the shear strength, setting time, compressive strength, and hydration process of the slurry, while maintaining desirable flowability. In this study, an orthogonal test was used to explore the effects of admixtures on the performance of the MCSF64-based slurries. The flow time, rheological parameters, rheological model, initial setting time, shear strength, compressive strength, pH variation, shrinkage/expansion, and microstructure of the MCSF64-based slurries were assessed. The optimal mix proportion of the MCSF64-based slurry was determined using Taguchi–Grey relational analysis. Through this study, some theoretical basis and guidance for the future application of low-pH grouting materials in the HLRW field may be provided.

## 2. Design Requirements and Materials

### 2.1. Design Requirements of Low-pH Grouting Materials

Table 1 summarizes the fundamental performance criteria of low-pH grouting materials [18,19]. The primary objective of these materials is to maintain a pH ≤ 11 to prevent any undesirable effects on the bentonite. Furthermore, the grout slurry must exhibit sufficient permeability to be injected into microcracks of at least 100 μm in size. In addition, the marsh funnel viscosity (flow time) should not exceed 45 s to ensure a desirable fluidity. Adequate strength is also required to withstand any outward water pressure following pre-grouting, and this can be achieved through effective work procedures and the management of early shear strength development. It should be noted that Table 1 gives good grouting experience guidance for laboratory determinations in HLRW around the world, but the exact criteria must be examined further, and these requirements are subject to verification by international research groups.

### 2.2. Materials

**Figure 1:**
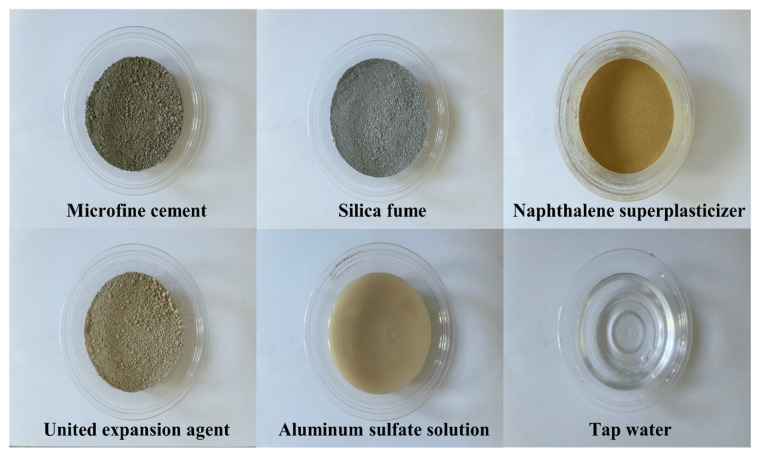
Composition design of raw materials used for MCSF64-based grouts.

**Figure 2:**
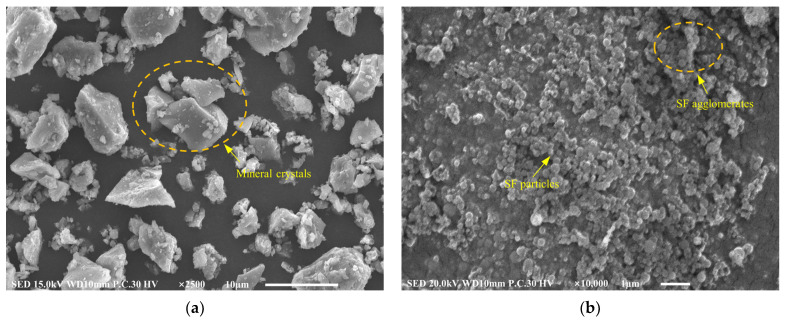
SEM images of raw materials: (**a**) MC; (**b**) SF.

**Figure 3:**
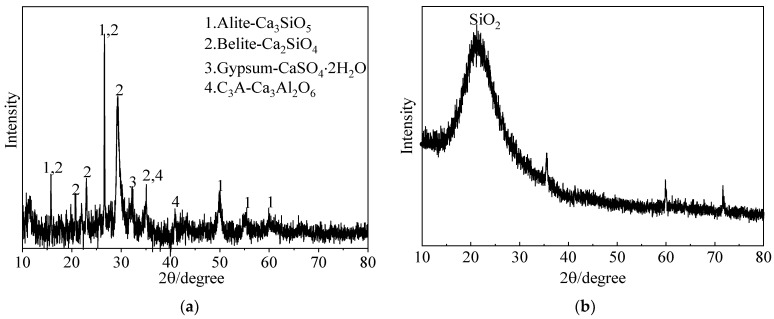
XRD spectra of raw materials: (**a**) MC; (**b**) SF.

The MC used in this study was the K1000 type of P.O 52.5 Portland cement (Qing Yun Kang Jing Building Materials Co., Ltd., Dezhou, China). Its performance was confirmed to adhere to the Chinese standard GB/T 35161-2017 [24]. The undensified SF was produced by Bo Run Building Materials Co., Ltd., Zhengzhou, China. The UEA (Wuhan Jianfeng Hongda Building Materials Co., Ltd., Wuhan, China) with a low alkalinity content was used to enhance the expansion performance of hardened grouts. The UEA conformed with Chinese standard GB/T 23439-2017 [25]. The details of the chemical components above are shown in Table 2, which was measured using X-ray fluorescence (XRF) (S8 Tiger, Bruker Technology Co., Ltd, Beijing, China)). NSP, one of the naphthalene sulfonated-formaldehyde copolymers, was produced by Chen Qi Co., Ltd. in Shanghai, China, and has a pH value of 8.00 (20 °C). In this study, a 50% alkali-free accelerator based on aluminum sulfate (AS) (Shanxi Feike New Materials Technology Co., Ltd., Yuncheng, China) was used. Table 3 presents the basic physical and chemical properties of AS provided by the manufacturer. The MCSF64-based slurry was prepared using tap water, and the designed raw materials of the slurry are shown in Figure 1. The SEM images of MC and SF, presented in Figure 2, show that SF particles are mostly spherical, and some groups of agglomerates could be observed, while the shape of the MC particles was angular and irregular. The X-ray diffraction (XRD) spectra (Smartlab9, Rigaku Corporation, Tokyo, Japan)) of MC and SF, which are presented in Figure 3, indicate that SF is primarily composed of amorphous silica. Furthermore, the SF has weak impurity peaks, which implies the presence of impurities in the SF. As can be seen from the XRD spectra, alite and belite were the main mineral phases in MC.

## 3. Methods

### 3.1. Preparation of Fresh Slurries and Samples

**Figure 4:**
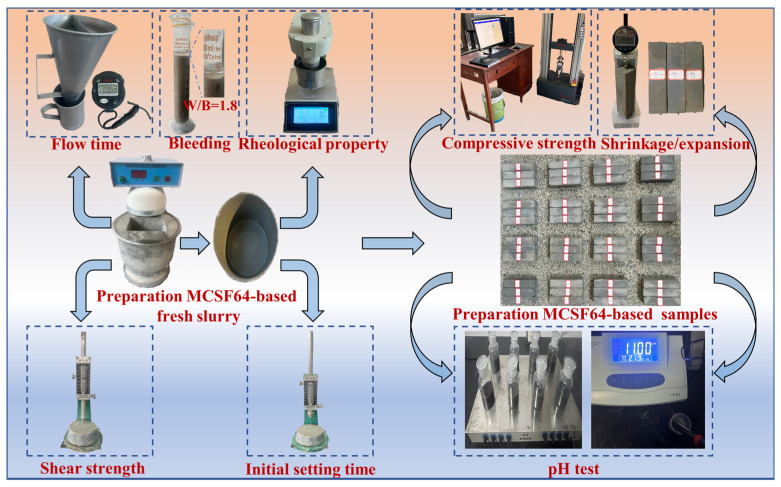
The sample preparation and test process of MCSF64-based slurry.

In this study, the fresh slurry was prepared in a similar method to that described by Posiva [19]. A high-speed mixer operating at 1500 r/min was used in a specific order: Firstly, water and NSP were added to the mixer, followed by the addition of MC, which was then quickly and uniformly stirred for 1 min. Next, AS and UEA were incorporated and the mixture was further agitated for 2 min. Finally, SF was added and stirred for an additional 2 min, with a total mixing time of approximately 5 min. After mixing, the slurry was poured into 40 mm × 40 mm × 160 mm molds and covered with a preservative film to prevent water evaporation. To ensure the maintenance of adequate moisture content, the surrounding area was continuously misted. After 2 days, the samples were demolded and cured under standard conditions of 20 ± 1 °C and 98% RH. To minimize the influence of temperature, slurry preparation and performance tests were carried out at room temperature. The detailed procedures for sample preparation and the testing of various properties are presented in Figure 4.

### 3.2. Experimental Methods of the MCSF64-Based Materials

Based on the performance requirements outlined in Table 1, the main properties of the MCSF64-based low-pH grouting materials investigated in this paper include pH value, particle size, flow time, plastic viscosity, yield stress, initial setting time, shear strength, and compressive strength. Bleeding capacity was not studied, because it was tested and observed in the laboratory that even with a W/B ratio of 1.8, the bleeding capacity of the MCSF64-based slurry remained below 5% after 2 h from preparation, indicating that the slurry could be considered stable according to SL62-2014 and EN 12715 standards. The test process is depicted in Figure 4, and the specific test methods for each property of the grouting materials are as follows.

**Figure 5:**
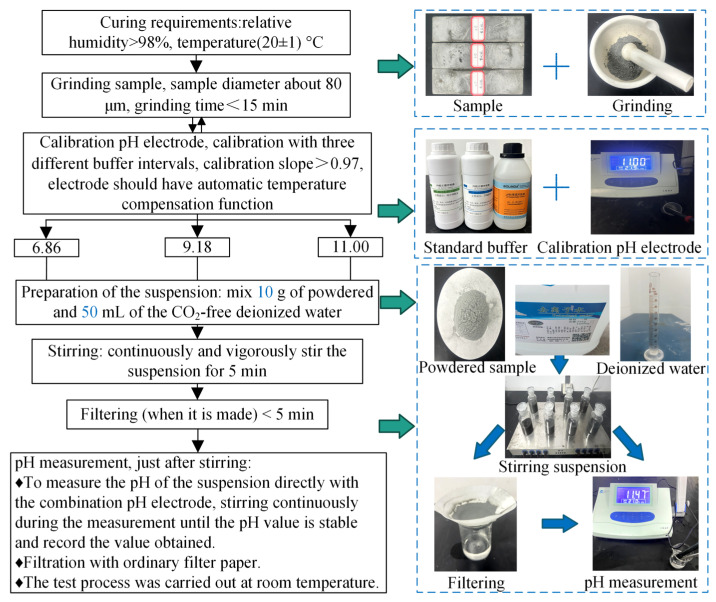
The pH test procedures of the S-ESL.

pH test: The measurement of pH in low-pH cementitious materials is a critical aspect of material characterization, particularly in the context of HLRW’s construction applications. The routine ex-situ leaching (R-ESL) method has been established as the standard method for this purpose by the Spanish National Research Council (CSIC) [26]. Recent comparisons between R-ESL and the pore fluid expression (PFE) method have demonstrated that R-ESL exhibits high levels of accuracy, repeatability, and reproducibility. However, the 1:1 solid to liquid ratio utilized in R-ESL can result in difficulties during filtrate extraction and lower test efficiency due to the requirement of nitrogen bubbling. To address these issues, a simplified ex-situ leaching (S-ESL) method was used in this study to measure the pH of grouts. The solid to liquid ratio was increased to 1:5 in S-ESL, thereby enabling the extraction of more filtrate. Moreover, the dilution during the preparation of the suspension was insufficient to desaturate the solution with respect to the C-S-H phase, and the measured pH was representative of the pore solution [27]. The experiments were conducted in an air-conditioned room with a controlled temperature of 21 ± 1 °C. The pH of the samples was measured using a PHS-3CB digital display acidity meter (Shanghai Yueping Scientific Instrument Co., Ltd., Shanghai, China). Figure 5 illustrates the test procedures of S-ESL.

Particle size: The particle size distributions of MC and SF were measured by a laser particle size analyzer (Bettersize2600, Dandong Baite Instrument Co., Ltd., Dandong, China).

Flow time (FT): The Marsh funnel viscometer is a straightforward apparatus characterized by a simple structure and easy operation, which makes it a viable option for assessing the viscosity changes of slurry in the engineering field. The manufacture of the Marsh funnel viscometer meets the American Petroleum Institute (API) standard. The methodology involves timing the flow of a 1500 mL slurry into a 946 mL measuring cup and recording the FT in seconds [28]. Before the actual test, water was used to calibrate the instrument (26 ± 0.5 s). The measurement was performed three times, and the average value was used to determine the final results.

Rheological property: The shear stress at various shear rates of the slurry was measured using a FANN-D12L digital rotating viscometer (Qingdao Senxin Co., Ltd., Qingdao, China). FANN-D12L is a rotational coaxial–cylindrical-type viscometer with twelve standard rotation speeds (i.e., 600, 300, 200, 100, 60, 30, 20, 10, 6, 3, 2 and 1 rpm). The relationship between shear rate (γ) and rotation speeds (*n*) is *γ* = 1.703 × *n* [29,30]. The corresponding shear rate is varied from 1021.8 s^−1^ to 1.703 s^−1^. At each rotation speed, the initial viscosity values (0 min after preparation) were tested, and then the slurry rheological model and rheological parameters, including plastic viscosity (PV) and yield stress (YS), were determined by fitting the test data.

Setting time: A Vicat apparatus was used to test the setting time of the fresh slurry corresponding to the Chinese Standard GB/T 1346-2011. In this study, only the initial setting time (IST) was used as an example to illustrate the coagulation characteristics of MCSF64-based grouts.

Shear strength (SS): In accordance with ISO 17892-6 and consistent with Posiva [18,19], the SS of fresh slurry after curing for 6 h was determined using a modified Vicat meter. The method involved the use of a cone with an angle of 30°, and the mass of the cone plus rod and index mark was 80 g. Each group comprised two specimens and was placed in the curing room after being prepared. In order to ensure the uniformity of the samples, the cone falling depth was recorded at three different positions for each sample, with a total of six data points being collected. The average value of these measurements was taken as the final result. The formula for calculating SS is as follows:

(1) $$\tau =\frac{cmg}{i^{2}}$$

where *τ* is shear strength, kPa; *m* is the mass of the cone, g; *g* is gravity acceleration, the value of which is 9.81 m/s^2^; *i* is fall cone depth, mm; *c* is a constant, which is related to the cone angle *β* (*β* = 30°, *c* = 0.80; *β* = 60°, *c* = 0.27).

Unconfined compression strength (UCS): The UCS of the hardened slurry was tested using an MTS CMT4304 (Shenzhen Meters Co., Ltd., Guangdong, China) with a servo-controlled system, and the sample was loaded continuously and evenly at a rate of 1.0 mm/min. To ensure the precision and reliability of the experimental results, six samples were tested for each group, and the average values were calculated.

Shrinkage/expansion: The shrinkage/expansion characteristics of hardened grouts were evaluated in accordance with JT/G603-2004 using a length comparometer. Specifically, three specimens, each of 40 mm × 40 mm × 160 mm, were demolded after 3 days in the standard curing room, and their initial length was measured at room temperature, with this time being designated as the testing zero point. In this study, one group was placed in a room with RH 30%, while another group was placed in water with RH 100%. From the date of sample forming, we recorded the daily length change after 4, 7, 14, 28, 56 and 90 days, respectively. The shrinkage/expansion ratio of grouts is calculated as follows:

(2) $$\varepsilon =\frac{L_{0}-L_{t}}{L-L_{d}}$$

where *L*_0_ is the initial length after curing for 3 d, mm; *L*_t_ is the length of the corresponding age, mm; *L* is sample length, 160 mm, and *L_d_* is the sum of the length of two copper nails embedded in the sample, 20 mm.

Morphology observation: After curing the samples for the designated period, the surface layer was removed, and the middle portion of each sample was crushed and collected. The fractured pieces were then immersed in absolute ethyl alcohol to halt the hydration process, and subsequently dried in an oven at 60 °C until reaching a constant weight. The morphology of the hydration products in the samples was investigated using a ZEISS GeminiSEM 360 scanning electron microscope manufactured by Carl Zeiss AG.

### 3.3. Taguchi and GRA Method

Orthogonal experimental design is a method used to study the effects of multiple factors and levels. This approach involves selecting representative points from comprehensive experiments based on orthogonality. These points possess the characteristics of “uniform dispersion, uniformity, and comparability”. By reasonably selecting the main factors that affect slurry performance and their corresponding levels, and then performing statistical analysis, the best combination of levels for different factors that achieve a specific goal can be determined. As a result, an orthogonal test was performed in this study to investigate the multi-objective parameters of MCSF64-based slurry mix proportions in order to find the best scheme. 

Grey relational analysis (GRA) is an available technique that leverages grey system theory to address multi-output problems with complex interrelationships. In terms of the GRA method, experimental data are first normalized between zero and one, and this is followed by a step referred to as grey relational generation. Subsequently, the grey relational coefficient, representing the correlation between the actual and desired experimental data, is determined by normalizing the data. The overall grey relational grade for each response is then calculated by averaging the estimated grey coefficients. Consequently, a multi-response problem can be transformed into a process optimization problem with the grey relational grade (GRG) serving as the objective function. Notably, some scholars have successfully applied the Taguchi method in combination with GRA to optimize the multi-objective proportioning of grouting materials, and this approach has demonstrated both feasibility and practicality [31].

By employing the Taguchi design method, experimental results are transformed into a signal-to-noise (S/N) ratio, which provides information about the degree of dispersion around the desired outcomes. The S/N ratio encompasses three types of performance characteristics, including lower-the-better, nominal-the-better, and higher-the-better. For this study, FT, PV, YS, and IST are categorized as small indicators (lower-the-better), whereas SS and UCS are classified as large indicators (higher-the-better), for MCSF64-based slurry. The S/N ratios for these three characteristics could be computed using Equations (3)–(5):

(3) $$\mathrm{Lower}\;-\;\mathrm{the}\;\mathrm{better}:\;S/N=-10\log_{10}\left(\frac{1}{n}\sum_{i=1}^{n}y_{i}{}^{2}\right)$$

(4) $$\mathrm{Nominal}\;-\;\mathrm{the}\;\mathrm{better}:\;S/N=-10\log_{10}\left(\frac{1}{n}\sum_{i=1}^{n}{\left(y_{i}-y_{0}\right)}^{2}\right)$$

(5) $$\mathrm{Higher}\;-\;\mathrm{the}\;\mathrm{better}:\;S/N=-10\log_{10}\left(\frac{1}{n}\sum_{i=1}^{n}{\left(\frac{1}{y_{i}}\right)}^{2}\right)$$

where *y_i_* is the observed value of the *i*th trial and *j*th repetition response, and *n* denotes the number of trials.

(6) $$Z_{ij}=\frac{\max\left(y_{ij}\right)-y_{ij}}{\max\left(y_{ij}\right)-\min\left(y_{ij}\right)};i=1,2,\ldots ,m;j=1,2,\ldots ,n$$

Equation (6) is chosen for the normalized S/N ratio in the lower-the-better case.

(7) $$Z_{ij}=\frac{|y_{ij}-\mathrm{Target}|-\min\left(|y_{ij}-\mathrm{Target}|\right)}{\max\left(|y_{ij}-\mathrm{Target}|\right)-\min\left(|y_{ij}-\mathrm{Target}|\right)};i=1,2,\ldots ,m;j=1,2,\ldots ,n$$

Equation (7) is chosen for the normalized S/N ratio in the nominal-the-better case.

(8) $$Z_{ij}=\frac{y_{ij}-\min\left(y_{ij}\right)}{\max\left(y_{ij}\right)-\min\left(y_{ij}\right)};i=1,2,\ldots ,m;j=1,2,\ldots ,n$$

Equation (8) is chosen for the normalized S/N ratio in the higher-the-better case.

(9) $$\Delta =\left(Z_{\max}-Z_{ij}\right);i=1,2,\ldots ,m;j=1,2,\ldots ,n$$

The quality loss function is computed using Equation (9).

(10) $$GRC_{ij}=\frac{\min(\Delta )+\lambda \max(\Delta )}{\Delta_{ij}+\lambda \max(\Delta )};i=1,2,\ldots ,m;j=1,2,\ldots ,n$$

The grey relational coefficient (*GRC*) is computed using Equation (10).

(11) $$GRG_{i}=\overset{n}{\underset{j=1}{\sum }}\varphi_{j}GRC_{ij};i=1,2,\ldots ,m$$

The grey relational grade (*GRG*) is computed using Equation (11).

where:

*y_ij_* is the S/N ratio value of the *i*th experiment for the *j*th response;

Z*_max_* is the optimal performance value of the *j*th characteristic;

Z*_ij_* is the *i*th normalized value of the *j*th characteristic;

Δ*_ij_* is the difference between the optimum value of the normalized S/N ratio and the *i*th normalized S/N ratio value for the *j*th response;

λ is the distinguishing coefficient (0 ≤ λ ≤ 1), and λ can be adjusted by the analyzer according to the practical needs, usually set as 0.5;

*GRC_ij_* is *GRC* for the *i*th replicate of *j*th response, *i* = 1, 2, …, m and *j* = 1, 2, …, *n*;

*φ_j_* is the weight of the *j*th response, and the sum of all *φ_j_* is equal to 1.0. All the responses (characteristics) considered in this research are given equal weighting.

According to the methodology mentioned above, the Taguchi experimental design method was utilized to select four factors, namely, W/B ratio, NSP content, AS content, and UEA content. After conducting extensive exploratory trials, the corresponding levels of the four selected factors were identified and are listed in Table 4. The content of UEA was determined by the manufacturer, who recommends that the content of UEA should be between roughly 6% and 8% by mass of cement. Therefore, the UEA content in the orthogonal test ranged from 3.6% to 4.8% by mass of binder. The L16 (4^5^) orthogonal test scheme was employed for the experiment, as shown in Table 5.

## 4. Results and Discussion

### 4.1. Grain Size Analysis

**Figure 6:**
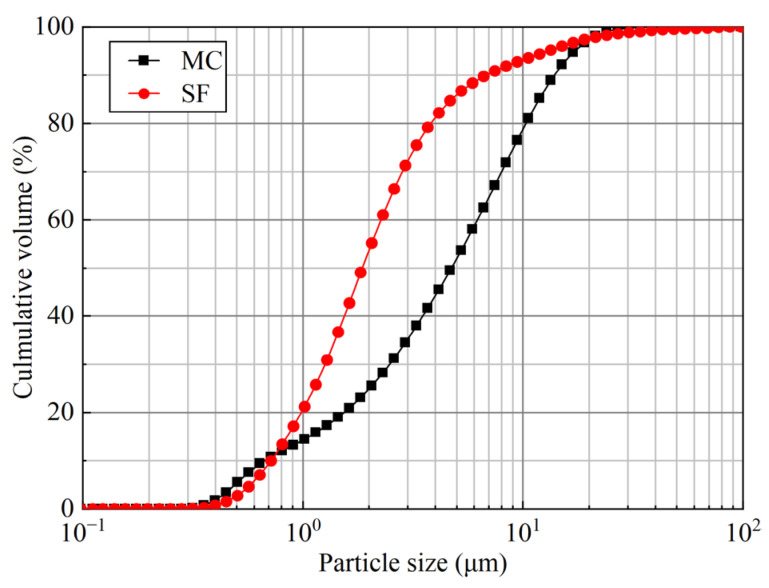
Particle size distributions of MC and SF.

Groutability is a critical parameter that must be considered when evaluating the effectiveness of injection grouts. The ability of the grouting material to permeate and propagate through rock fractures is influenced by various factors, including the geometrical parameters of the fracture, as well as the particle size, rheological properties, and stability of the grouting material. It has been widely observed that grouting materials with smaller particle sizes exhibit increased injectability [32]. The particle size distribution of the raw materials utilized in this study is depicted in Figure 6, and Table 6 presents the characteristic grain sizes and specific surface areas of MC and SF. Data analysis indicates that both MC and SF meet the requirements of EN 12715, which specifies a specific surface area greater than 800 m^2^/kg and *d*_95_ less than 20 μm. It is worth noting that the actual particle size of SF is smaller than that of MC, while the particle size distribution of SF is relatively wide, which suggests that this material tends to agglomerate, despite attempts to reduce agglomeration via the ultrasonic treatment of the powder. This phenomenon has also been reported by Wei et al. [33]. In practice, the relationship between the particle size of cement and fracture aperture can be assessed using the “groutability ratio”, which is defined as follows [34]:

(12) $$G=b/d_{95}$$

where *G* is the groutability ratio, *b* is the aperture of fracture (μm), and *d*_95_ is 95% by volume of the particle diameter smaller than this value. Penetration ability is considered difficult if *G* < 2 and satisfactory if *G* > 5. In this study, *G* = 100 μm/15.05 μm ≈ 6.6. Thus, satisfactory permeation can be achieved.

### 4.2. Analysis of the Taguchi–Grey Relational and Variance Results

#### 4.2.1. Flow Time of the MCSF64-Based Slurry

**Figure 7:**
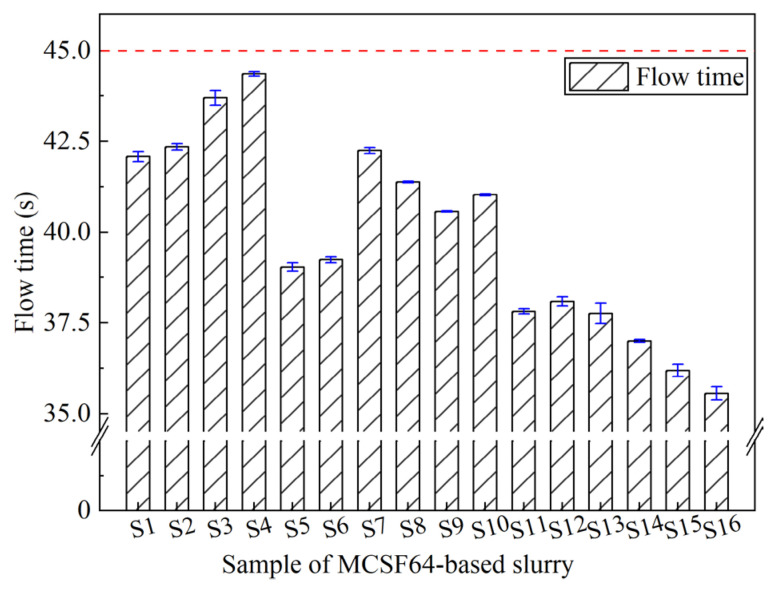
Flow time of the MCSF64-based slurry.

The evaluation of the fluidity and workability of fresh slurry heavily depends on the FT. Typically, slurries with smaller FT values tend to have better groutability and spread ability. Figure 7 displays the FT results for fresh slurries (S1 to S16). As demonstrated in Figure 7, all fresh slurries satisfied the requirements specified in Table 1, where the FT was less than 45 s. The FT of the slurries varied between 35.56 s and 44.36 s. It is noteworthy that S4, which had a 1.4 W/B ratio, 1.9% NSP, 4.8% AS, and 4.8% UEA, exhibited the highest FT among all MCSF64-based slurries. Obviously, the W/B ratio had a significant impact on FT, and S16 had a higher W/B ratio, as well as lower AS and UEA contents, hence showing the lowest FT.

In Table 7, the signal-to-noise (S/N) ratios given by FT, YS, PV, and IST are calculated using Equation (3), while SS and 28-day UCS are calculated using Equation (5). The average S/N ratio (ASNR) refers to the average value of all S/N ratio values at a certain level with a specific factor. In this paper, minitab26 was used to analyze the test data.

**Figure 8:**
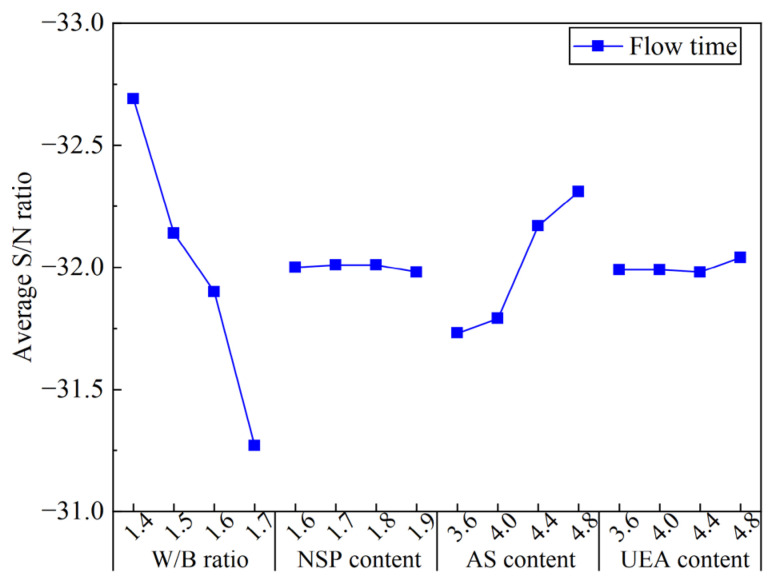
Average S/N ratio values of flow time of the MCSF64-based slurry.

Figure 8 presents the ASNR values for the FT of the MCSF64-based slurries for different factors. It can be seen from Figure 8 that there was a notable decrease in the ASNR values of the FT with an increase in the W/B ratio of the slurry from 1.4 to 1.7. This trend can be attributed to the increased presence of free water in the liquid phase and reduced friction between MC and SF particles. The ASNR values showed a moderate decline when the NSP content exceeded 1.8%, which can be explained by the introducing of a high amount of SO_4_^2−^ ions when adding AS. These ions weaken the adsorption degree and dispersion ability of NSP to MC particles, resulting in a minor variation in the FT from a macroscopic view. The addition of AS content results in a significant increase in FT, as the detrimental effect of AS on FT can be attributed as follows: (i) the main component of AS is aluminum sulfate (Al_2_(SO_4_)_3_), which enhances the hydration of tricalcium aluminate (C_3_A) and the initial dissolution of tricalcium silicate (C_3_S) after mixing, resulting in the rapid formation of acicular ettringite (AFt) crystals and the consumption of water; (ii) the adsorption of a large number of SO_4_^2−^ ions on MC particles will lead to a competitive adsorption effect with NSP anions, which diminishes the electrostatic repulsion of NSP. Therefore, the increase in AS remarkably weakens the fluidity and dispersibility of fresh slurry. Zhang et al. [35] also reported similar results. Moreover, these marked and regular trends reflect that the data obtained in FT tests are reliable, although previous studies have shown that the standard Marsh cone nozzle is not very sensitive in low-viscosity grouting materials [36]. The UEA content appeared to have little effect on the FT of the fresh slurry, with the ASNR showing a slight increase when the UEA content exceeded 4.4%.

#### 4.2.2. Rheological Model of the MCSF64-Based Slurry

**Figure 9:**
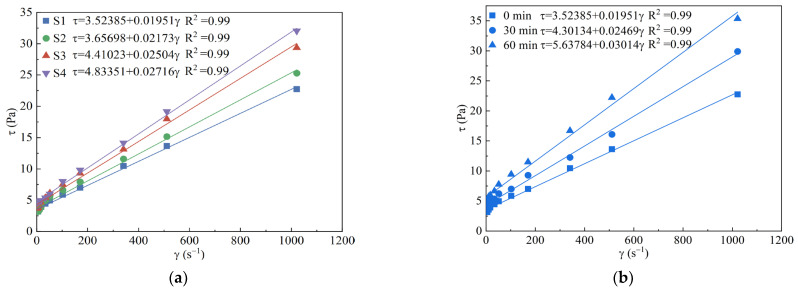
Rheological model and rheological parameters: (**a**) typical fitting curves (S1 to S4) of fresh slurry; (**b**) fitting curves of fresh slurry (S1) with elapsed time.

In terms of cement-based slurry, rheological properties are typically characterized by dividing the slurries into Newtonian fluid, Bingham fluid, and power law fluid categories based on the water–cement ratio [37]. However, incorporating 40% SF in binary low-pH grouting material may result in a rheological model that differs from the conventional cement-based slurry. Usually, the rheological parameters of slurry can be obtained by fitting different rheological constitutive equations on the diagram of shear stress (*τ*) and shear rate (*γ*), which is helpful in evaluating the rheological properties of MCSF64-based slurries. Figure 9a shows the test data and fitting curves of fresh MCSF64-based slurry (S1 to S4).

Based on the data presented in Figure 9a, the relationship between the *γ* and *τ* of all fresh slurries can be approximated with a straight line. The experimental data were fitted using the Bingham fluid equation, and in most cases, the resulting correlation coefficients (R^2^) were equal to 0.99. This suggests that the rheological model of MCSF64-based slurry can be considered as Bingham fluid, and hence, the rheological parameters can be obtained after fitting data:

(13) $$\tau =\tau_{0}+\eta \gamma$$

where *τ* is the shear stress, Pa; *τ*_0_ is the yield stress, Pa; *η* is the plastic viscosity, Pa·s; *γ* is the shear rate, s^−1^.

As shown in Figure 9b, the rheological behavior of S1 followed a Bingham model over a period of 0 to 60 min, with γ fixed at 100 rpm (170.3 s^−1^). During this period, the yield stress (YS) of S1 increased by 160%, ranging from 3.52 Pa to 5.64 Pa, while the plastic viscosity (PV) increased by 154%, ranging from 19.51 mPa·s to 30.14 mPa·s. The increasing effect on the rheological parameters is mainly accounted for by the hydration reaction, especially when the C_3_A of the fresh slurry begins, resulting in the free water decreasing and the sediment gradually increasing, hence the increase in rheological parameters with time [38].

#### 4.2.3. Rheological Parameters of the MCSF64-Based Slurry

**Figure 10:**
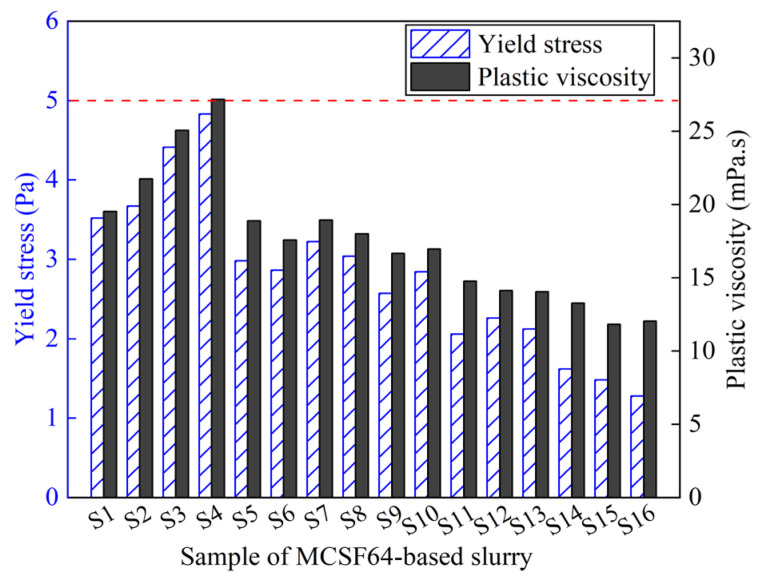
Plastic viscosity and yield stress of the MCSF64-based slurry.

To acquire a satisfactory grouting effect, the rheological parameters of the slurry should be as low as possible to maintain superior flowability and groutability. In this regard, the PV and YS of MCSF64-based fresh slurries were investigated, as shown in Figure 10. The results indicate that the rheological parameters of all fresh slurries examined met the requirements outlined in Table 1. Notably, mix S4 demonstrated the highest YS of 4.83 Pa and PV of 27.16 mPa·s, while mix S16 exhibited the lowest YS of 1.28 Pa and a relatively lower PV of 12.02 mPa·s. This behavior can be attributed to the dominant effect of the W/B ratio and AS content, with mix S16 containing a higher W/B ratio and lower AS content compared to mix S4.

**Figure 11:**
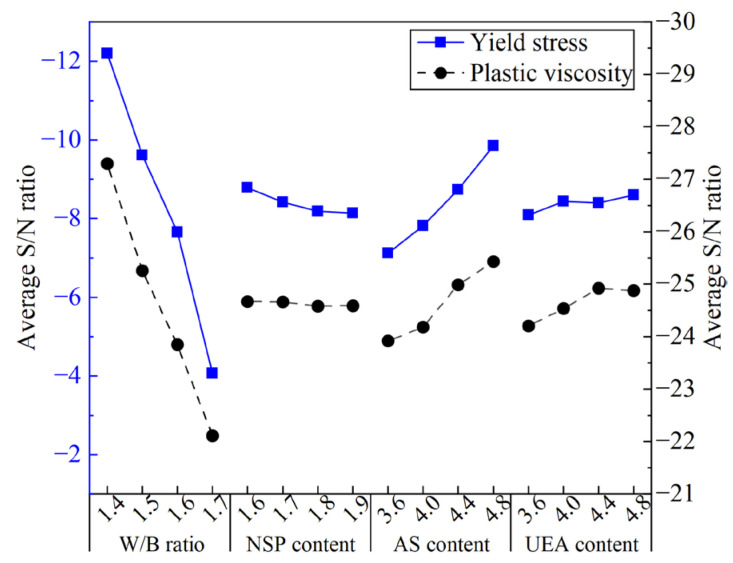
Average S/N ratio values of yield stress and plastic viscosity of the MCSF64-based slurry.

Figure 11 shows the ASNR values for YS and PV in the MCSF64-based slurry for different factors. As shown in Figure 11, the variation of the W/B ratio on YS exhibited a similar trend to that of PV. Specifically, the ASNR decreased dramatically as the W/B ratio increased, which can be attributed to the higher amount of free water leading to lower internal friction force and a greater lubrication effect on MC and SF particles. The ASNR of YS and PV decreased as the NSP content increased. This observation is attributable to the fact that NSP, being an anionic surfactant, can directionally adsorb onto the surface of MC particles, lowering the solid–liquid interface energy and forming a double-electron layer solvation membrane via electrostatic repulsion. As a result, the flocculation structure caused by the high surface energy of MC and SF begins to decompose into smaller ones, releasing more free liquid, and improving the fluidity of fresh slurry. It is noted that the variation in the mean S/N ratio of PV is not visible compared to YS. A possible reason for this phenomenon is that the change in PV was small due to the uniform dispersing of particles in suspensions, thus the effect of NSP on PV is less remarkable. The available literature [39] also reports similar results. The ASNR of YS and PV increased with a small increase in the AS content. This is because AS can react with C_3_A or Ca(OH)_2_ to form AFt, and a large amount of free water is consumed in the formation process of AFt, which increases the internal friction within hydration products, leading to an increase in the values of the rheological parameter of fresh slurry. The variation in the ASNR presented a rising trend as UEA content increased, which indicates that the addition of UEA increased the rheological parameter’s values by consuming a part of the water in the slurry.

#### 4.2.4. Initial Setting Time of the MCSF64-Based Slurry

**Figure 12:**
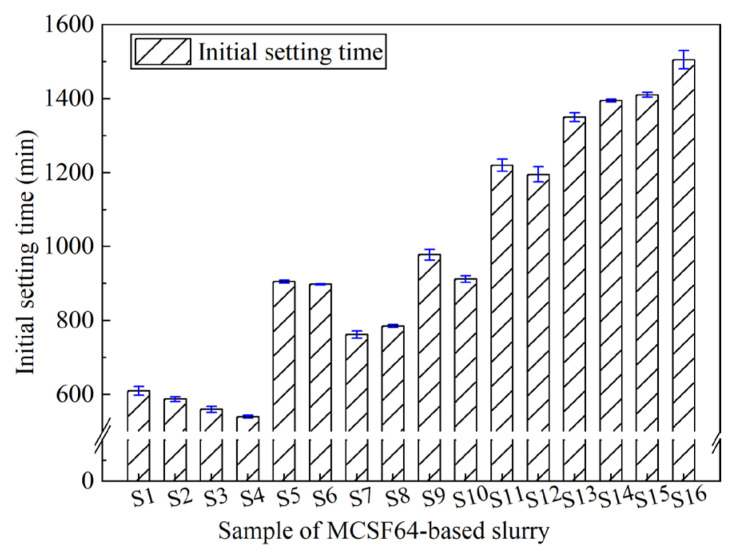
Initial setting time of the MCSF64-based slurry.

In grouting, careful control of the slurry’s setting time is essential to achieve optimal diffusion radius and effect. According to previous studies, it is appropriate to control the final setting time within 4–24 h [40]. Figure 12 displays the initial setting time (IST) of MCSF64-based slurries, which ranges from 540 to 1505 min. Among tested slurries, mix S16 has the longest IST due to its higher W/B ratio and lower AS content. Conversely, mix S4 has the shortest IST due to the dominant influence of the W/B ratio and higher AS and UEA contents compared to other slurries.

**Figure 13:**
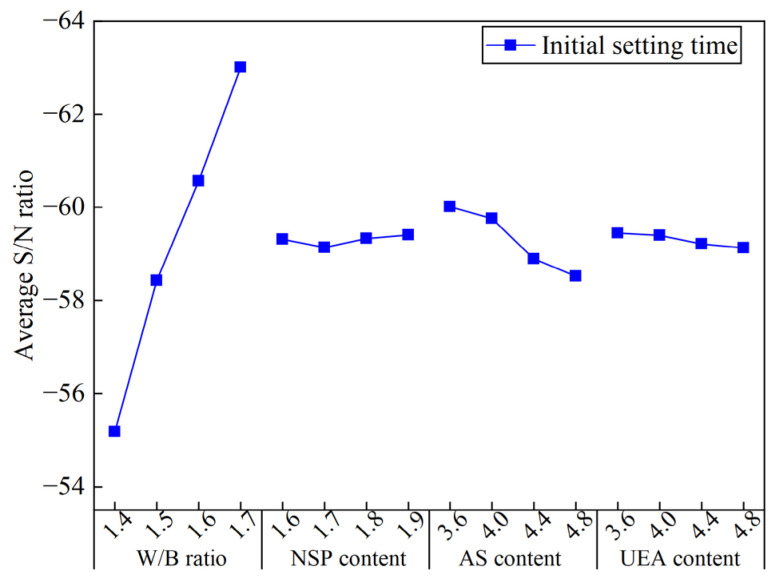
Average S/N ratio values of initial setting time of the MCSF64-based slurry.

Figure 13 shows the ASNR values of the IST for MCSF64-based slurries with different factors. As shown in Figure 13, the ASNR of the IST increased as the W/B ratio increased from 1.4 to 1.7. The increase in the W/B ratio can cause a decrease in the cement concentration (particularly C_3_A and C_3_S), and subsequently slow down the hydration process of the cement. Therefore, the IST was prolonged. The ASNR increased with an increase in the NSP content, indicating that the inclusion of NSP in the slurries prolongs the IST to a certain extent by adsorbing its molecules on nucleating hydrate particles, which inhibits the development of hydration products and lowers the efficient hydration reaction in the liquid phase. Similar observations have been reported by Wang et al. [41]. The ASNR decreases notably with an increase in the AS content. The above phenomenon can be explained as follows: when a liquid Al_3_SO_4_ is added to the fresh slurry, a possible reaction between cement and Al_3_SO_4_ can be expressed by Equations (14)–(17) [42]. Moreover, the reaction in Equation (15) is more likely to happen at early ages because of the extra addition of Al^3+^ and SO_4_^2−^ by AS; thereby, the rapid formation of AFt crystals shortens the IST of fresh slurry. This observation is in good agreement with those in previously published studies [43]. The variation in the ASNR values fell moderately with UEA content. UEA is a type of sulfoaluminate expansion agent composed mainly of CaO, SO_3_ and Al_2_O_3_. The addition of UEA during cement hydration leads to water consumption and the formation of AFt, which can slightly reduce the IST of MCSF64-based slurries [44]. However, due to the powdery nature of UEA, its hydration rate is slower when compared to AS. Consequently, the effect of UEA on the IST is not as pronounced as that of AS.

(14) $${\mathrm{Al}}^{3+}+4{\mathrm{OH}}^{-}⇌\mathrm{Al}{\left(\mathrm{OH}\right)}_{3}+{\mathrm{OH}}^{-}⇌{\left[\mathrm{Al}{\left(\mathrm{OH}\right)}_{4}\right]}^{-}\;$$

(15) $$2[\;{\mathrm{Al}{\left(\mathrm{OH}\right)}_{4}]}^{-}+6{\mathrm{Ca}}^{2+}+4{\mathrm{OH}}^{-}+3{\mathrm{SO}}_{4}^{2-}+26H_{2}O⇌C_{3}\mathrm{AA}\cdot 3{\mathrm{CaSO}}_{4}A\cdot 32H_{2}O$$

(16) $$4\;{\left[\mathrm{Al}{\left(\mathrm{OH}\right)}_{4}\right]}^{-}+6{\mathrm{Ca}}^{2+}+8{\mathrm{OH}}^{-}+C_{3}\mathrm{AA}\cdot 3{\mathrm{CaSO}}_{4}A\cdot 32H_{2}O⇌3C_{3}\mathrm{AA}\cdot {\mathrm{CaSO}}_{4}A\cdot 12H_{2}O+8H_{2}O$$

(17) $$2\;{\left[\mathrm{Al}{\left(\mathrm{OH}\right)}_{4}\right]}^{-}+4{\mathrm{Ca}}^{2+}+4{\mathrm{OH}}^{-}+{\mathrm{SO}}_{4}^{2-}+6H_{2}O⇌C_{3}\mathrm{AA}\cdot {\mathrm{CaSO}}_{4}A\cdot 12H_{2}O$$

#### 4.2.5. Shear Strength of the MCSF64-Based Slurry

**Figure 14:**
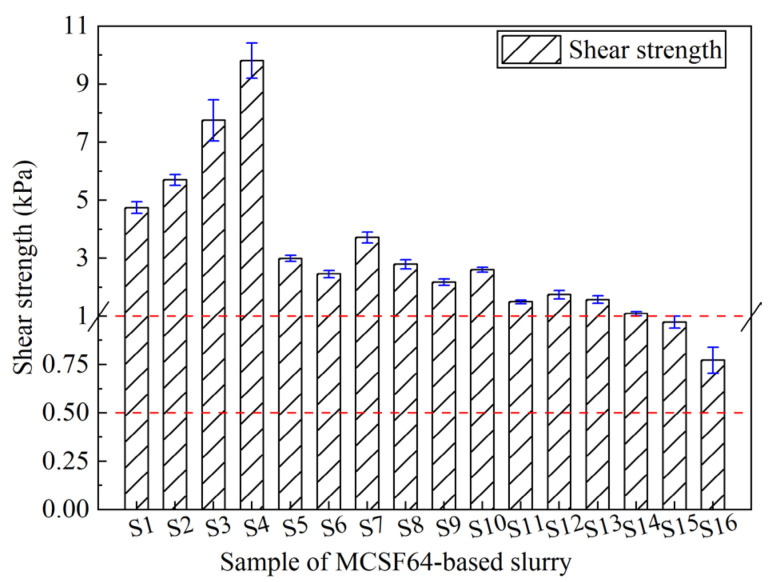
Shear strength of the MCSF64-based slurry.

The characterization of internal deformation and flow resistance of slurry is a crucial aspect that hinges on the measurement of SS. In the context of water plugging, early SS plays a more pivotal role than compressive strength. Figure 14 illustrates the SS of the MCSF64-based slurry after curing for 6 h. The achieved properties of all the slurry samples, as per Table 1, satisfied the desired standards (6 h > 0.5 kPa). Mix S4 demonstrated the highest SS of 9.81 kPa among all the slurries studied, while mix S16 recorded the lowest SS values of 0.77 kPa, with corresponding falling depths of 8 mm and 28.5 mm, respectively. The wide variance observed in the SS of the MCSF64-based slurries is attributable to the prominent impact of the W/B ratio and AS content.

**Figure 15:**
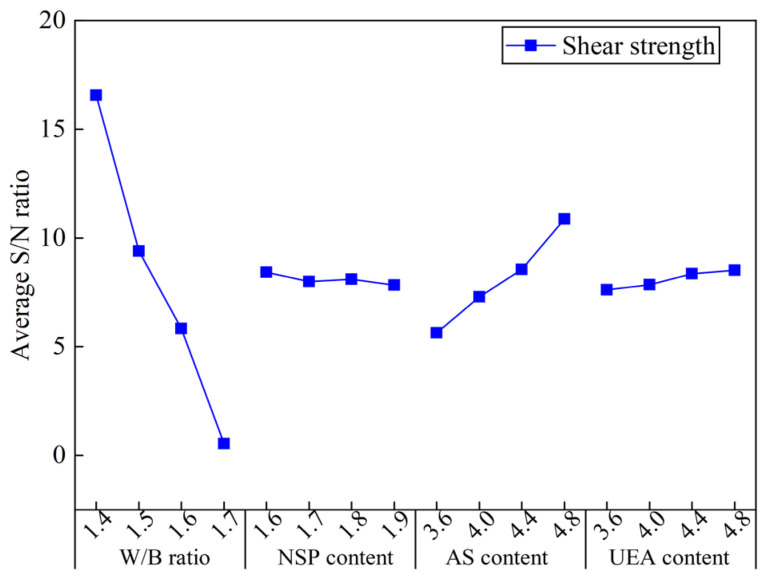
Average S/N ratio values of shear strength of the MCSF64-based slurry.

The ASNR values of SS for all slurries with different factors are illustrated in Figure 15. Obviously, the shorter the setting time of the slurry, the higher the corresponding SS. Therefore, those factors that lead to a shorter setting time, such as a low W/B ratio, high AS and UEA content, enhance the early SS of the slurry. Section 4.2.4 provides a detailed explanation of this mechanism. Additionally, the acidic environment created by AS facilitates the dissolution of C_3_S, promoting the hydration reaction of cement at early stages [45]. This, in turn, fosters the growth of AFt, which acts as a skeleton and, along with C-S-H gels and other hydration products, directly contributes to the development of the early SS of the slurry. 

#### 4.2.6. Unconfined Compression Strength of the MCSF64-Based Slurry

**Figure 16:**
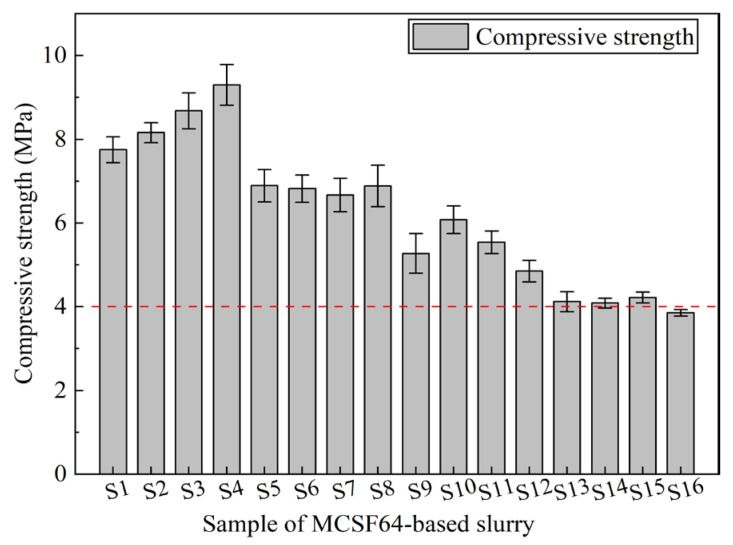
28-day UCS of the MCSF64-based slurry.

The long-term stability of grouting materials and the reinforcement effect of surrounding rock are believed to be positively correlated with the compressive strength of hardened slurry. The 28-day UCS of hardened MCSF64-based slurries is depicted in Figure 16. As shown in Figure 16, all hardened slurry samples, except for mix S16 (3.86 MPa), exceeded 4 MPa. Since the W/B ratio has a marked effect on UCS, S4 exhibited the highest UCS of 9.86 MPa. Furthermore, slurries with a higher AS content demonstrated relatively higher values than those with a lower AS content. Hence, the W/B ratio and AS content are inferred to be crucial factors in the UCS development of MCSF64-based slurries. Based on the findings of this study, it is recommended that the W/B ratio of MCSF64-based slurries does not exceed 1.7.

**Figure 17:**
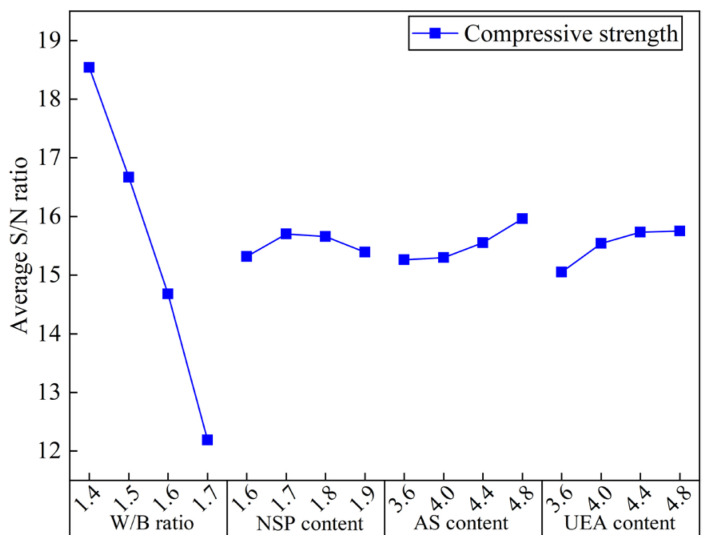
Average S/N ratio values of the 28-day UCS of the MCSF64-based slurry.

The ASNR values of the 28-day UCS of the hardened MCSF64-based slurries for different factors are presented in Figure 17. The results indicate that increasing the W/B ratio from 1.4 to 1.7 causes a significant decrease in ASNR, primarily due to the elevated total porosity and inferior pore structure in hardened grouts, resulting in a lower UCS in MCSF64-based slurries. This observation complies with previously reported research [46]. The ASNR trend shows an initial rise followed by a decline with increasing NSP content, indicating that an optimal NSP dosage of 1.7% may be most effective in achieving a higher UCS after a 28-day curing period. A reasonable explanation is that the effect of NSP on the UCS of hardened MCSF64-based slurries is not significant, with the 28-day UCS depending mainly on the W/B ratio and AS content. The ASNR of the 28-day UCS increases significantly with AS content, which can be attributed to the introduction of Al^3+^ and SO_4_^2−^ by AS, reducing the concentration of Ca(OH)_2_ produced by C_3_S and C_2_S and leading to the formation of AFt. Furthermore, SO_4_^2−^ can replace SiO_4_^4−^ in the C–S–H gel, enabling free SiO_4_^4−^ to react with the outer layer Ca^2+^ and form C–S–H gel, thereby promoting cement hydration and enhancing the UCS of the slurries [47]. The addition of UEA can also increase the UCS of hardened grouts, potentially due to the loose AFt layer that allows Ca^2+^ to pass through the network microstructure and react with highly activated minerals (SiO_2_ and Al_2_O_3_) from UEA. This reaction also consumes Ca(OH)_2_ and accelerates the hydration process of the cement, while the increase rate of ASNR values of UCS becomes gentle when its content exceeds 4.4%.

#### 4.2.7. Procedure of Optimal Grout Mix Selection Using the Taguchi–Grey Relational Analysis Method

MCSF64-based slurries containing various additives with different properties can be optimized by analyzing and discussing the orthogonal test results presented above. Nevertheless, the optimal level for specific performance is not necessarily the best combination to satisfy all the attributes. Therefore, based on Taguchi-based Grey relational analysis, idealized levels for different properties were obtained in the present study.

In accordance with the Taguchi–Grey relational analysis method described in Section 3.3, the S/N ratio, summarized in Table 7, was normalized using Equations (6) and (8). Then, the quality loss functions were computed using Equation (9) based on the normalized data. At last, the *GRC* values were computed using Equation (10). Then, these *GRC* values were subsequently converted into a single Grey relational grade (*GRG*) employing Equation (11). The resultant *GRC* values and *GRG* are displayed in Table 8.

Table 8 presents data indicating that mix S4, characterized by a W/B ratio of 1.4, 1.9% NSP, 4.8% AS, and 4.8% UEA, demonstrated the highest *GRG* value of 0.6667. The mix S4 exhibited the lowest IST, the highest early SS, and a 28-day UCS that surpassed all other groups. Nonetheless, mix S4’s fluidity was poor, as indicated by its high FT and rheological parameters, making it debatable as to whether it is the optimal mix. In order to identify the best combination of MCSF64-based slurries, the mean *GRG* for each parameter level was calculated. The maximum average *GRG* for every parameter level had a significant effect on the responses. The mean *GRG* values for all parameters are provided in Table 9, where the maximum mean *GRG* values for each factor are denoted (*).

As per the data presented in Table 9, the optimal combination for an MCSF64-based slurry was found to be the mix with a W/B ratio of 1.4, 1.9% NSP, 3.6% AS, and 4.8% UEA, which obtained the highest mean *GRG* values. Based on the above discussion, it can be inferred that the optimal mix can meet all the design requirements of low-pH grouting materials.

#### 4.2.8. Analysis of Variance (ANOVA)

**Figure 18:**
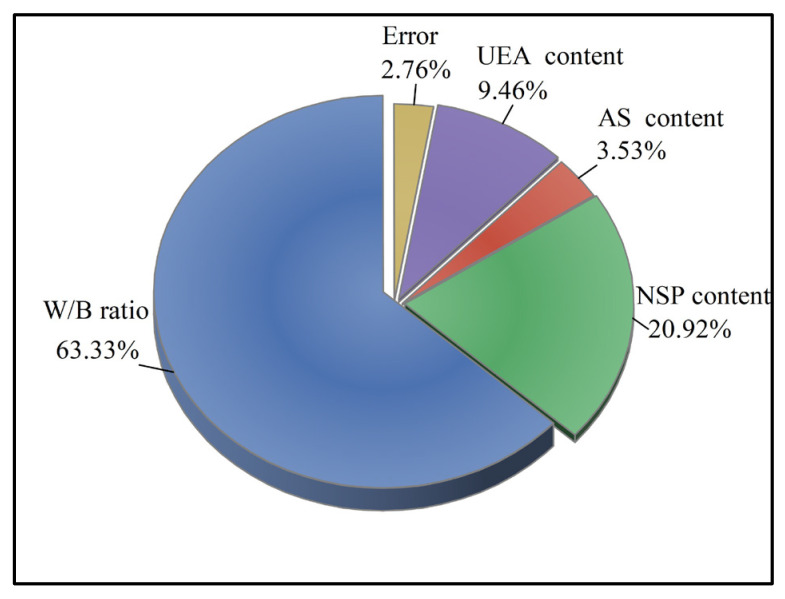
Percentage contributions of the four factors in the *GRG*.

In this study, ANOVA was utilized to determine the degree of effect of each factor on the MCSF64-based slurries, specifically to assess the percentage contribution of four factors toward the results of the *GRG*. Table 10 presents the ANOVA results of the *GRG* for all the factors, while Figure 18 illustrates the percentage contributions of the four factors on the tests. Based on the findings of Table 10 and Figure 18, it can be inferred that the W/B ratio was the most critical factor, with the highest contributing effect of 63.33%. NSP also shows a high contribution ratio (20.92%) because NSP plays a crucial role in improving slurry fluidity. Moreover, the order of percentage contribution for the four factors was W/B ratio > NSP content > UEA content > AS content.

#### 4.2.9. Comparison between the Optimal Mix and the Reference Mix

This section presents a comparative analysis between the properties of an optimal mix and a reference mix (W/B ratio of 1.4, 1.6% NSP) without AS and UEA additives [23]. The optimal mix, obtained by applying Taguchi–Grey relational analysis, consists of a W/B ratio of 1.4, 1.9% NSP, 3.6% AS, and 4.8% UEA. The results of the tests, which are provided in Table 11, indicate that the optimal mix possesses superior properties in terms of SS, IST, and UCS, while maintaining a desirable fluidity that meets the design requirements outlined in Table 1. Specifically, the optimal mix exhibits SS values of 3.2 kPa and 4.02 kPa at 6 h and 8 h, respectively, whereas the reference mix shows 0 kPa at these times. Moreover, the IST with a measured 632 min of the optimal mix decreases by nearly 50% compared to the reference mix. Furthermore, the UCS values of the optimal mix at 4 days and 28 days are 1.16 MPa and 8.77 MPa, respectively, showing increases of 139.7% and 116.9% compared to the reference mix. Additionally, since AS and UEA are inorganic additives, their impact on the groundwater environment is minimal. Therefore, the findings suggest that the optimal mix can serve as a viable candidate for use as a low-pH grouting material in HLRW.

### 4.3. pH of Hardened Optimal Mix and Reference Mix

**Figure 19:**
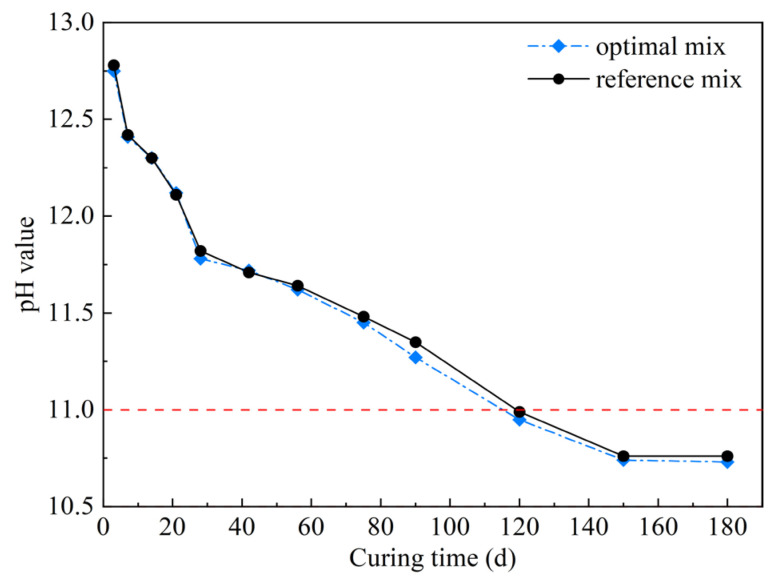
Variation in pH values of pore solutions of different grout mixes with curing time.

Figure 19 shows plots of the pH variation versus curing time. As shown in Figure 19, the pH values for the optimal mix and reference mix were approximately 12.75–12.78 after curing for 3 day. This observation can be interpreted as implying that, on the one hand, the pozzolanic reaction between SF and CH formed during cement hydration begins to occur after 3 days [33,48]. On the other hand, the pore solution, primarily composed of Na^+^, K^+^, Ca^2+^ and OH^−^, had a high ionic strength, resulting in a relatively higher pH value. As time went by, the pH values of two different mixes gradually decreased, and finally converged to 10.74–10.76 after about 150 days. The target pH value was obtained after curing for 120 days. In addition to this, it is observed that the pH value of the optimal mix is slightly lower when compared with the reference mix. This is mainly because the formation of AFt reduces the Ca^2+^ concentration, hence the measured pH value is comparatively decreased. The pH variation of the reference mix in this study is similar to that in previous investigations [11]. The mechanism of pH reduction with curing time can be explained by the following factors [8,12,49]: (i) the MC is diluted, lowering the alkali and hydroxide concentrations in the pore solution significantly; (ii) the CH is consumed by the pozzolanic reaction, leading to lower Ca^2+^ concentration in the pore solution; (iii) the additional C–S–H gel with a low Ca/Si ratio formed by the reaction of the SF with CH enhanced its alkali absorption capacity, which in turn reduced the equilibrium pH.

### 4.4. Shrinkage and Expansion Analysis

**Figure 20:**
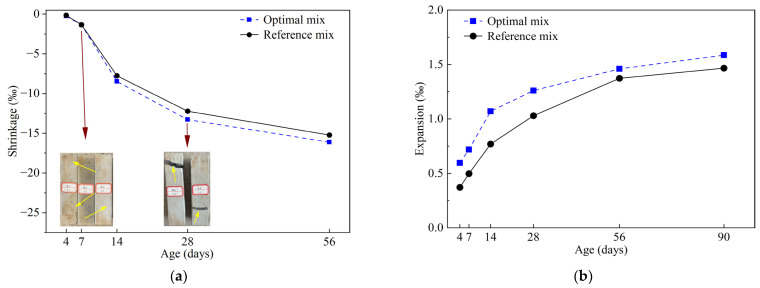
Shrinkage and expansion rates of optimal mix and reference mix with age: (**a**) shrinkage rate; (**b**) expansion rate.

The results of shrinkage and expansion for the two mixes are presented in Figure 20. The optimal mix and reference mix experienced shrinkages when placed in dry environments with RH 30%, with respective rates of 0.25–16.14‰ and 0.16–15.24‰. This was anticipated as injection grout materials possess a high W/B ratio, rendering mixes susceptible to drying shrinkage triggered by physical water loss. In addition, the movement of water molecules away from capillaries (5–50 nm) through evaporation in dry conditions is another reason for the shrinkage of hardened grouts [50,51]. The optimal mix exhibited slightly higher shrink rates than the reference mix over time, attributed to the former generating more AFt and consuming more internal water under the same conditions. Microcracks appeared on the outer surfaces of both mixes after 7 days due to intensive water loss. Furthermore, at 28 days (perhaps earlier), two-thirds of the samples in the two mixes showed interconnectivity cracks, and only one sample could still be used for testing. A similar crack phenomenon of MCSF64 hardened grouts was observed by Orantie and Kuosa [23]. However, it should be highlighted that injection grouts are seldom exposed to RH below 30% in underground engineering, and the groundwater will likely prevent the grout inside sealed fissures from being subjected to water depletion, and thus drying. As shown in Figure 20b, the expansion rate (0.59–1.59‰) of the optimal mix remained higher than that of the reference mix (0.37–1.47‰), increasing by 159% and 122% at 4 and 28 days, respectively. This implies that the expansion rate was less pronounced than the shrinkage rate, and the optimal mix had a superior expansion capacity compared to the reference mix. Generally, micro-expansion will produce a certain degree of compressive stress, which improves the tightness between grouting materials and rock mass. Indeed, the addition of AS and UEA in the optimal mix had a synergistic effect on forming AFt, thereby enhancing its early expansion ability. Specifically, the generation of AFt by adding UEA in the early stage can be achieved by following Equation (18). In the later period, the KAl_3_(SO_4_)_2_**·**(OH)_6_ from UEA may provide a stable expansion source due to its slower hydration, followed by Equation (19) [52]. Moreover, these findings confirm that grouts require water within a certain time to finish the hydration, and not just for a short period after preparation [53].

(18) $$3CaO\cdot 3Al_{2}O_{3}\cdot CaSO_{4}+6Ca(OH{)}_{2}+8CaSO_{4}+90H_{2}O\to 3(CaO\cdot Al_{2}O_{3}\cdot 3CaSO_{4}\cdot 32H_{2}O)$$

(19) $$2KAl_{3}{\left(SO_{4}\right)}_{2}\cdot {\left(OH\right)}_{6}+13Ca{\left(OH\right)}_{2}+5CaSO_{4}+78H_{2}O\to 3(3CaO\cdot Al_{2}O_{3}\cdot 3CaSO4\cdot 32H_{2}O)+2KOH$$

### 4.5. Microstructure Analysis of the Hardened Slurries

**Figure 21:**
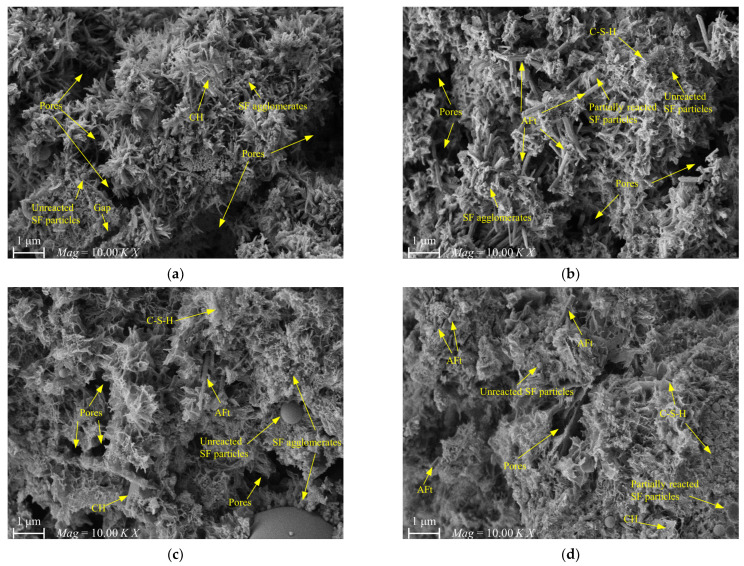
SEM images of hardened slurry at different curing ages: (**a**) reference mix at 4-day; (**b**) optimal mix at 4-day; (**c**) reference mix at 28-day; (**d**) optimal mix at 28-day.

Figure 21 presents the SEM images of the reference and optimal mixes after 4 and 28 days of hydration. As illustrated in Figure 21a,b, both mixes exhibited inadequate hydration processes after 4 days of curing, resulting in a loose and porous microstructure in the hydration products. Moreover, the internal microstructure of the hardened slurry displayed unreacted SF particles, SF agglomerates, and larger voids. The incorporation of 40% SF in the mix resulted in a “dilution effect” that decreased the concentration of MC and delayed the generation of the C-S-H gel. In addition, the pozzolanic reaction between SF and Ca(OH)_2_ was weak during the early stages of hydration, and SF particles mainly served as physical fillers. Thus, the two mixes showed a lower hydration degree after curing for 4 days. Particularly, it can be seen from Figure 21b that due to the addition of AS and UEA, the acicular AFt crystals were crosslinked and intercalated, and extended to the edges of the pores in the hydration products of the optimal mix, leading to a more compact space in the optimal hardened slurry, which decreased the amounts of macropores and increased the early strength and expansion capacity when compared with the reference mix.

Figure 21c,d show the microstructures of the two mixes after an extended curing period of 28 days, showing a relatively higher degree of hydration reaction, with a reduction in gap and pore sizes between hydration products and a denser microstructure of the primary binding phase. The SEM images also indicate that most SF particles have a non-clean and non-smooth surface, with numerous hydration products covering the surfaces of the spherical particles, all of which collectively indicates that more C-S-H gels with a low Ca/Si ratio were generated. The freshly produced dense gel firmly wraps around the particle surface and develops a strong connection with the surrounding hydration products, resulting in the reduced porosity and refined pore size distribution of the hardened slurry, as well as higher UCS. Specifically, compared to the SEM images of the reference mix after 28 days of curing, Figure 21d reveals more needle-like AFt crystals, which is conducive to improving the structural density and reducing the pore shrinkage stress of the hardened grouts to some extent. These findings suggest that the optimal mix has a higher expansion rate than the reference mix, which aligns with the expansion test results.

## 5. Conclusions

Based on the results presented in this paper, the following conclusions can be derived:

(1) The orthogonal test results and variance analysis of the Grey correlation grade show that the degree of influence on the properties of MCSF64-based slurries was as follows—W/B ratio > NSP content > UEA content > AS content;
(2) For the rheological curves of MCSF64-based slurry, the Bingham model was suitable for predicting the rheological properties because the correlation coefficients were 0.99 in all cases, and the Bingham model remained constant from 0 min to 60 min;
(3) The addition of AS and UEA positively affected the shear strength, initial setting time, and compressive strength of the MCSF64-based slurries, while reducing the fluidity of fresh slurry. However, the detrimental effects of AS and UEA on the fluidity of slurry can be negated by adding NSP;
(4) The addition of AS and UEA accelerated the hydration process, decreased the pH value, enhanced the expansion ability, and improved the microstructure of the MCSF64-based slurries compared with slurries without AS and UEA;
(5) The results of the Taguchi–Grey relational analysis show that the optimal mix proportion of the MCSF64-based slurry was found to be a W/B ratio of 1.4, 1.9% NSP, 3.6% AS, and 4.8% UEA, which can satisfy the design requirements proposed by Posiva.

## Figures and Tables

**Table 1:** Requirements used for the development of low-pH grouting materials.

Order of Importance	Property	Requirement [18]	Updated Requirement [19]
Required Properties	pH	≤11	≤11
	Penetration ability b_min_	≤80 μm	≤80 μm
	Penetration ability b_crit_	≤120 μm	≤120 μm
Desired Properties	Plastic viscosity	≤50 mPa.s	≤50 mPa.s
	Yield stress	≤5 Pa	≤5 Pa
	Bleed	≤10%	≤2%
	Workability time	≥60 min	≥60 min
	Shear strength	6 h ≥ 500 Pa	6 h ≥ 500 Pa
	Compressive strength	28 d ≥ 4 MPa	28 d ≥ 4 MPa
	Marsh funnel viscosity (Flow time)	-	≤45 s

**Table 2:** The main chemical components of raw materials.

Material	SiO_2_	CaO	Al_2_O_3_	Fe_2_O_3_	MgO	SO_3_	Na_2_O	K_2_O	TiO_2_	P_2_O_5_	LOI
MC (wt. %)	21.10	61.60	4.08	3.95	3.64	2.40	0.18	0.35	0.40	0.16	2.14
SF (wt. %)	96.30	0.30	0.50	0.12	0.20	0.12	0.10	0.31	-	0.35	1.70
UEA (wt. %)	38.01	18.85	12.93	4.69	5.34	17.15	0.13	0.71	0.21	-	1.74

**Table 3:** Basic physical and chemical properties of accelerators.

Accelerator	Solid Content/%	Alkali Content/%	Cl^−^ Content/%	F^−^ Content/%	pH Value
AS	50.63	0.05	0.02	0.04	2.2

**Table 4:** Four factors and four levels of MCSF64-based orthogonal test.

Level	Factors			
	W/B Ratio	NSP Content (%)	AS Content (%)	UEA Content (%)
1	1.4	1.6	3.6	3.6
2	1.5	1.7	4.0	4.0
3	1.6	1.8	4.4	4.4
4	1.7	1.9	4.8	4.8

**Table 5:** The MCSF64-based slurry orthogonal test scheme L16 (4^5^).

Sample	W/B Ratio	NSP Content (%)	AS Content (%)	UEA Content (%)	Blank
S1	1(1.4)	1(1.6)	1(3.6)	1(3.6)	1
S2	1(1.4)	2(1.7)	2(4.0)	2(4.0)	2
S3	1(1.4)	3(1.8)	3(4.4)	3(4.4)	3
S4	1(1.4)	4(1.9)	4(4.8)	4(4.8)	4
S5	2(1.5)	1(1.6)	2(4.0)	3(4.4)	4
S6	2(1.5)	2(1.7)	1(3.6)	4(4.8)	3
S7	2(1.5)	3(1.8)	4(4.8)	1(3.6)	2
S8	2(1.5)	4(1.9)	3(4.4)	2(4.0)	1
S9	3(1.6)	1(1.6)	3(4.4)	4(4.8)	2
S10	3(1.6)	2(1.7)	4(4.8)	3(4.4)	1
S11	3(1.6)	3(1.8)	1(3.6)	2(4.0)	4
S12	3(1.6)	4(1.9)	2(4.0)	1(3.6)	3
S13	4(1.7)	1(1.6)	4(4.8)	2(4.0)	3
S14	4(1.7)	2(1.7)	3(4.4)	1(3.6)	4
S15	4(1.7)	3(1.8)	2(4.0)	4(4.8)	1
S16	4(1.7)	4(1.9)	1(3.6)	3(4.4)	2

**Table 6:** Gradation of MC and SF.

Materials	Grain Sizes (μm)				Specific Surface (m^2^/kg)
	*d*_95_	*d*_90_	*d*_50_	*d*_10_	
MC	14.22	11.59	4.23	0.85	998
SF	15.05	7.66	2.09	0.81	1327

**Table 7:** S/N ratio values of different properties of the MCSF64-based slurry.

Sample	FT	YS	PV	IST	SS	28-Day UCS
S1	−32.4815	−10.9309	−25.8051	−55.7066	13.5291	17.7860
S2	−32.5371	−11.2933	−26.7412	−55.3875	15.1094	18.2338
S3	−32.8096	−12.8888	−27.9727	−54.9638	17.7873	18.7704
S4	−32.9398	−13.6789	−28.6786	−54.6479	19.8334	19.3697
S5	−31.8302	−9.4843	−25.5108	−59.1330	9.5134	16.7644
S6	−31.8746	−9.1273	−24.8855	−59.0655	7.7922	16.6757
S7	−32.5165	−10.1571	−25.5384	−57.6391	11.3992	16.4803
S8	−32.3358	−9.6575	−25.0958	−57.8974	8.9121	16.7539
S9	−32.1641	−8.1987	−24.4335	−59.8068	6.7390	14.4390
S10	−32.2620	−9.0664	−24.5834	−59.1999	8.3328	15.6781
S11	−31.5544	−6.2773	−23.3758	−61.7272	3.4868	14.8702
S12	−31.6162	−7.0822	−22.9967	−61.5474	4.8068	13.7148
S13	−31.5406	−6.5267	−22.9350	−62.6067	3.9158	12.2979
S14	−31.3640	−4.1903	−22.4443	−62.8915	0.7485	12.2274
S15	−31.1718	−3.4052	−21.4523	−62.9844	−0.2646	12.5062
S16	−31.0192	−2.1442	−21.5981	−63.5507	−2.2368	11.7205

**Table 8:** *GRC* values of different properties of all the grout mixes.

Sample	*GRC* Values						Grey Relational Grade	*GRG* Order
	FT	YS	PV	IST	SS	28-Day UCS		
S1	0.3964	0.3963	0.4536	0.8079	0.6364	0.7072	0.5663	6
S2	0.3875	0.3866	0.4059	0.8575	0.7002	0.7710	0.5848	5
S3	0.3491	0.3493	0.3566	0.9337	0.8436	0.8645	0.6161	4
S4	0.3333	0.3333	0.3333	1.0000	1.0000	1.0000	0.6667	1
S5	0.5421	0.4400	0.4710	0.4981	0.5167	0.5948	0.5105	10
S6	0.5289	0.4523	0.5128	0.5019	0.4782	0.5867	0.5101	11
S7	0.3907	0.4185	0.4693	0.5981	0.5668	0.5696	0.5022	14
S8	0.4218	0.4343	0.4979	0.5780	0.5026	0.5938	0.5047	13
S9	0.4562	0.4879	0.5479	0.4632	0.4573	0.4368	0.4749	16
S10	0.4359	0.4545	0.5357	0.4944	0.4897	0.5088	0.4865	15
S11	0.6421	0.5825	0.6526	0.3860	0.4030	0.4595	0.5210	8
S12	0.6166	0.5387	0.7006	0.3922	0.4234	0.4035	0.5125	9
S13	0.6481	0.5682	0.7090	0.3587	0.4094	0.3510	0.5074	12
S14	0.7358	0.7381	0.7846	0.3506	0.3664	0.3487	0.5540	7
S15	0.8629	0.8206	1.0000	0.3481	0.3544	0.3578	0.6240	3
S16	1.0000	1.0000	0.9612	0.3333	0.3333	0.3333	0.6602	2

**Table 9:** Mean *GRG* values for each level of all the factors.

Factor	Level 1	Level 2	Level 3	Level 4	Max.–Min.
W/B ratio	0.6085 *	0.5069	0.4987	0.5864	0.0221
NSP content	0.5148	0.5339	0.5658	0.5860 *	0.0202
AS content	0.5644 *	0.5579	0.5374	0.5407	0.0269
UEA content	0.5338	0.5295	0.5683	0.5689 *	0.0352

*: optimal level for each factor.

**Table 10:** ANOVA results of *GRG* for the factors.

Factor	DOF ^1^	SOS ^2^	MS ^3^	F Value	*p* Value	Percentage Contribution/%
W/B ratio	3	0.0369	0.0123	22.9323	0.0143	63.33
NSP content	3	0.0122	0.0041	7.5736	0.0652	20.92
AS content	3	0.0021	0.0007	1.2771	0.4227	3.53
UEA content	3	0.0055	0.0018	3.4242	0.1695	9.46
Error	3	0.0016	0.0005	-	-	2.76
Total	15	0.0583				100.00

^1^ DOF: Degree of freedom; ^2^ SOS: sum of squares; ^3^ MS: average squares.

**Table 11:** The test results between the optimal mix and the reference mix.

Different Mix	Properties								
	Bleeding/%	FT/s	YS/Pa	PV/mPa.s	6 h SS/kPa	8 h SS/kPa	IST/min	4-Day UCS/MPa	28-Day UCS/MPa
Optimal mix	0.2	42.36	2.92	19.85	3.20	4.02	632	1.16	8.77
Reference mix	0.5	40.92	2.08	18.14	0	0	1265	0.83	7.50

## Data Availability

The data presented in this study are available on request to the corresponding author.

## References

[1] Wang J., Chen L., Su R., Zhao X. (2018). The Beishan Underground Research Laboratory for Geological Disposal of High-Level Radioactive Waste in China: Planning, Site Selection, Site Characterization and in Situ Tests. J. Rock Mech. Geotech. Eng..

[2] Vasconcelos R.G., Walkley B., Day S., Tang C.C., Paraskevoulakos H., Gardner L.J., Corkhill C.L. (2020). 18-Month Hydration of a Low-pH Cement for Geological Disposal of Radioactive Waste: The Cebama Reference Cement. Appl. Geochem..

[3] Duro L., Altmaier M., Holt E., Mäder U., Claret F., Grambow B., Idiart A., Valls A., Montoya V. (2020). Contribution of the Results of the CEBAMA Project to Decrease Uncertainties in the Safety Case and Performance Assessment of Radioactive Waste Repositories. Appl. Geochem..

[4] Savage D., Noy D., Mihara M. (2002). Modelling the Interaction of Bentonite with Hyperalkaline Fluids. Appl. Geochem..

[5] Lothenbach B., Le Saout G., Haha M.B., Figi R., Wieland E. (2012). Hydration of a Low-Alkali CEM III/B–SiO_2_ Cement (LAC). Cem. Concr. Res..

[6] Dauzères A., Le Bescop P., Cau-Dit-Coumes C., Brunet F., Bourbon X., Timonen J., Voutilainen M., Chomat L., Sardini P. (2014). On the Physico-Chemical Evolution of Low-pH and CEM I Cement Pastes Interacting with Callovo-Oxfordian Pore Water under Its in-Situ CO_2_ Partial Pressure. Cem. Concr. Res..

[7] Vogt C., Lagerblad B., Wallin K., Baldy F., Jonasson J.-E. (2009). Low-pH Self Compacting Concrete for Deposition Tunnel Plugs.

[8] Lothenbach B., Scrivener K., Hooton R. (2011). Supplementary Cementitious Materials. Cem. Concr. Res..

[9] Vollpracht A., Lothenbach B., Snellings R., Haufe J. (2016). The Pore Solution of Blended Cements: A Review. Mater. Struct..

[10] Rossen J.E., Lothenbach B., Scrivener K.L. (2015). Composition of C–S–H in Pastes with Increasing Levels of Silica Fume Addition. Cem. Concr. Res..

[11] Kim J.-S., Kwon S., Choi J.-W., Cho G.-C. (2011). Properties of Low-pH Cement Grout as a Sealing Material for the Geological Dis-posal of Radioactive Waste. Nucl. Eng. Technol..

[12] Coumes C.C.D., Courtois S., Nectoux D., Leclercq S., Bourbon X. (2006). Formulating a Low-Alkalinity, High-Resistance and Low-Heat Concrete for Radioactive Waste Repositories. Cem. Concr. Res..

[13] García Calvo J.L., Hidalgo A., Alonso C., Fernández Luco L. (2010). Development of Low-pH Cementitious Materials for HLRW Repositories. Cem. Concr. Res..

[14] Lothenbach B., Rentsch D., Wieland E. (2014). Hydration of a Silica Fume Blended Low-Alkali Shotcrete Cement. Phys. Chem. Earth Parts A/B/C.

[15] Savage D., Benbow S. (2007). Low-pH Cements.

[16] Güllü H., Cevik A., Al-Ezzi K.M.A., Gülsan M.E. (2019). On the Rheology of Using Geopolymer for Grouting: A Comparative Study with Cement-Based Grout Included Fly Ash and Cold Bonded Fly Ash. Constr. Build. Mater..

[17] Xiao R., Polaczyk P., Jiang X., Zhang M., Wang Y., Huang B. (2021). Cementless Controlled Low-Strength Material (CLSM) Based on Waste Glass Powder and Hydrated Lime: Synthesis, Characterization and Thermodynamic Simulation. Constr. Build. Mater..

[18] Sievaeen U., Syrjänen P., Ranta-aho S. (2005). Injection Grout for Deep Repositories-Low-pH Cementitious Grout for Larger Frac-Tures.

[19] Sievänen U., Raivio P., Vuorinen U., Hansen J., Norokallio J., Syrjänen P. (2006). Optimisation of Technical Properties of Low-pH Cementitious Injection Grout.

[20] Boden A., Sievaenen U. (2005). Low-pH Injection Grout for Deep Repositories.

[21] Hakanen M., Ervanne H. (2006). The Influence of Organic Cement Additives on Radionuclide Mobility.

[22] Holt E. (2008). Durability of Low-pH Injection Grout.

[23] Orantie K., Kuosa H. (2008). Durability 2007. Injection Grout Investigations. Background Description.

[24] Wang J., Wang Z., Tu Y. (2017). Ultra-fine Portland Cement.

[25] Zhao S., Liu L., Zhang X. (2017). Expansive Agents for Concrete.

[26] Alonso M.C., Garcia C., Walker C. (2012). Development of an Accurate pH Measurement Methodology for the Pore Fluids of Low-pH Cementitious Materials.

[27] Bach T., Coumes C.C.D., Pochard I., Mercier C., Revel B., Nonat A. (2012). Influence of Temperature on the Hydration Products of Low-pH Cements. Cem. Concr. Res..

[28] Guria C., Kumar R., Mishra P. (2013). Rheological Analysis of Drilling Fluid Using Marsh Funnel. J. Pet. Sci. Eng..

[29] Liu Q., Lei G., Peng X., Lu C., Wei L. (2018). Rheological Characteristics of Cement Grout and Its Effect on Mechanical Properties of a Rock Fracture. Rock Mech. Rock Eng..

[30] Li W., Shaikh F.U.A., Wang L., Lu Y., Wang B., Jiang C., Su Y. (2019). Experimental Study on Shear Property and Rheological Characteristic of Superfine Cement Grouts with Nano-SiO_2_ Addition. Constr. Build. Mater..

[31] Cui W., Huang J., Song H., Xiao M. (2017). Development of Two New Anti-Washout Grouting Materials Using Multi-Way ANOVA in Conjunction with Grey Relational Analysis. Constr. Build. Mater..

[32] Eriksson M., Friedrich M., Vorschulze C. (2004). Variations in the Rheology and Penetrability of Cement-Based Grouts—An Experimental Study. Cem. Concr. Res..

[33] Wei Y., Yao W., Xing X., Wu M. (2012). Quantitative Evaluation of Hydrated Cement Modified by Silica Fume Using QXRD, ^27^Al MAS NMR, TG–DSC and Selective Dissolution Techniques. Constr. Build. Mater..

[34] Xu S., Xing L., Wang G., Han Q. (2014). Experimental Research on Grouting of Superfine Cement Slurry in Micro-Fissured Rock Body. J. Saf. Sci. Technol..

[35] Zhang S., Qiao W., Wu Y., Fan Z., Zhang L. (2020). Multi-Response Optimization of Ultrafine Cement-Based Slurry Using the Taguchi-Grey Relational Analysis Method. Materials.

[36] Jorne F., Henriques F.M., Baltazar L.G. (2015). Injection Capacity of Hydraulic Lime Grouts in Different Porous Media. Mater. Struct..

[37] Güllü H. (2016). Comparison of Rheological Models for Jet Grout Cement Mixtures with Various Stabilizers. Constr. Build. Mater..

[38] Fu Y., Mei C., Chen X., Li W., Yu B., Li X., Wang B., Wang S. (2023). The Time-Dependent Grout Buoyancy Behavior Based on Cement Hydration Mechanism. Cem. Concr. Res..

[39] Yoshioka K., Tazawa E., Kawai K., Enohata T. (2002). Adsorption Characteristics of Superplasticizers on Cement Component Minerals. Cem. Concr. Res..

[40] Perret S., Palardy D., Ballivy G. (2000). Rheological Behavior and Setting Time of Microfine Cement-Based Grouts. Mater. J..

[41] Wang W., Yuan Y., Liang X., Qin Z., Chen Z., Ding K., Xia Y., Yan C. (2022). Experimental Study on Floor Damage and Slurry Material Ratio Optimization in Deep and High Confined Water Mining. Processes.

[42] Paglia C., Wombacher F., Böhni H. (2001). The Influence of Alkali-Free and Alkaline Shotcrete Accelerators within Cement Systems: I. Characterization of the Setting Behavior. Cem. Concr. Res..

[43] Salvador R.P., Cavalaro S.H., Segura I., Figueiredo A.D., Pérez J. (2016). Early Age Hydration of Cement Pastes with Alkaline and Alkali-Free Accelerators for Sprayed Concrete. Constr. Build. Mater..

[44] Guo J., Zhang S., Guo T., Zhang P. (2020). Effects of UEA and MgO Expansive Agents on Fracture Properties of Concrete. Constr. Build. Mater..

[45] Li G., Zhang J., Niu M., Song Z. (2020). The Mechanism of Alkali-Free Liquid Accelerator on the Hydration of Cement Pastes. Constr. Build. Mater..

[46] Hou Z., Tang M., Liang S., Zhu Y. (2021). Optimization of the Physical and Mechanical Properties of Grouting Material for Non-Soil-Squeezing PHC Pipe Pile. Crystals.

[47] Wang Y., Shi C., Ma Y., Xiao Y., Liu Y. (2021). Accelerators for Shotcrete–Chemical Composition and Their Effects on Hydration, Microstructure and Properties of Cement-Based Materials. Constr. Build. Mater..

[48] Qing Y., Zenan Z., Deyu K., Rongshen C. (2007). Influence of Nano-SiO_2_ Addition on Properties of Hardened Cement Paste as Compared with Silica Fume. Constr. Build. Mater..

[49] Hong S.-Y., Glasser F.P. (2002). Alkali Sorption by C-S-H and C-A-S-H Gels: Part II. Role of Alumina. Cem. Concr. Res..

[50] Li S., Sha F., Liu R., Zhang Q., Li Z. (2017). Investigation on Fundamental Properties of Microfine Cement and Cement-Slag Grouts. Constr. Build. Mater..

[51] Tran N.P., Gunasekara C., Law D.W., Houshyar S., Setunge S., Cwirzen A. (2021). A Critical Review on Drying Shrinkage Mitigation Strategies in Cement-Based Materials. J. Build. Eng..

[52] Liu Y., Chi Y., Tian W., Zhang J. (2022). Effect of Different Expanders on Properties of Cement-Based Grouting Material. J. Build. Mater..

[53] Mirza J., Saleh K., Langevin M.-A., Mirza S., Bhutta M.A.R., Tahir M. (2013). Properties of Microfine Cement Grouts at 4 °C, 10 °C and 20 °C. Constr. Build. Mater..

